# Society Meetings

**Published:** 1908-02

**Authors:** 


### SOCIETY MEETINGS.

---

The Fifth Pan-American Medical Congress will be held in Guatemala, Central America, August 5, 6, 7, 8, 9, and 10, 1908. The International executive committee consists of Dr. Charles A. L. Reed, president; Dr. A. Vander Veer, vice president; and Dr. Ramon Guiteras, secretary, together with 29 members mostly from South American countries. The president of the congress is Dr. Juan J. Orteaga, Guatemala, and the secretary general is Dr. Jose Azurdia, Guatemala.

The committee in its preliminary announcement says: it must not be thought that Guatemala is an undesirable place to visit in August and that it will be very hot and the rain constant. August is the time of the year called the cunicula, when, although hot there is but very little rain. The heat of Guatemala does not however compare with the heat of our own states, as it is situated on a plateau which is comparatively cool. The trip down from New Orleans or from New York by steamer to Porto Barrios is an agreeable one. The trip to the Congress and back will be in the hands of the Chairman of the Transportation Committee. Excursion trips can also be made in connection with this Congress to Mexico or the various West Indian Islands. The excursions will be in the hands of Thomas Cook & Co., or any one who chooses to organise one. There will be no charge for transportation in the Republic of Guatemala.

Members and prospective members of the congress from North America including the Dominion of Canada, who propose to attend or read papers, should communicate with Dr. Ramon Guiteras, Secretary of the International Executive Committee, 75 West 55th Street, New York.

---

THE third annual meeting of the Ohio Association of Medical Teachers was held in the afternoon and evening of December 27, 1907, and was the most successful in the history of the organisation. About seventy-five members were present besides a number of visitors, among them the presidents of several literary colleges of the state. The entire program was carried out with two exceptions. Interest in the organisation has increased and its members feel that much good has been accomplished.

---

THE Southern Surgical and Gynecological Association held its twentieth annual meeting at New Orleans, December 17, 18, and 19, 1907, under the presidency of Dr. Howard A. Kelly, of Baltimore. The three days were filled in the reading of papers and their discussions on interesting subjects, relating to gynecology and surgery. Officers were elected for the ensuing year as follows: president, F. W. Parham, New Orleans; vice presidents, Willis F. Westmoreland, Atlanta, and Henry D. Fry, Washington; secretary, William D. Haggard, Nashville, reëlected; treasurer, Stuart McGuire, Richmond. Saint Louis was chosen as the next place of meeting and John Young Brown of that city was appointed chairman of the committee of arrangements.

---

THE Medical Society of the State of New York is holding its one hundred and second annual meeting at Albany, while these pages are going through the press, under the presidency of Dr. Frederic C. Curtis, of that city. A large and interesting program is being disposed of and a full attendance is registered.

---

THE Buffalo Academy of Medicine held meetings during January as follows:

*Section on Surgery.*—Tuesday evening, January 7. Program: (*a*) A discussion of practical cystoscopy, with presentation of cystoscopes, examining catheterising and operative, Bransford Lewis, St. Louis; (*b*) Exhibition of lantern slides of skin and genitourinary diseases, David E. Wheeler.

*Section on Medicine.*—Tuesday evening, January 14, Program: Curative value of rest in children with chronic loss of appetite, Irving M. Snow.

*Section on Pathology.*—Tuesday evening, January 21. Program: Report of a case of Lymphatic Leukemia, A. E. Woehnert; Dr. Almuth Wright's laboratory and the practical application of his opsonic methods, W. Harry Glenny; Blood cultures, Charles A. Bentz.

The discussion was led by Edward J. Meyers.

---

THE Western Surgical and Gynecological Association held its seventeenth annual meeting at Saint Louis, December 30, and 31, 1907, at which time the following-named officers were elected for the ensuing year: president, W. W. Grant, Denver; vice presidents, Willard Bartlett, Saint Louis, and Harry A. Sifton, Milwaukee; secretary and treasurer, Arthur T. Mann, Minneapolis. The next meeting will be held at Minneapolis, December 30, and 31, 1908.

---

THE Syracuse Academy of Medicine held its annual meeting January 7, 1908. Dr. A. B. Miller, retiring president, delivered an address and Dr. F. C. Curtis, of Albany, president of the Medical Society of the State of New York, read a paper by invitation, entitled, "Smallpox and diseases confounded with it."

---

THE Elmira Academy of Medicine held its annual meeting Wednesday, January 8, 1908. The retiring president, Dr. H. W. Fudge, delivered the annual address and papers were read as follows: "The present status of opinion concerning the etiology of tuberculosis," by C. W. M. Brown; "Some considerations of systemic infection through diseased tonsils," by G. M. Case.

---

THE Rochester Academy of Medicine held its annual meeting January 24, 1908, at which the usual order of business was disposed of, including the election of officers.

